# Genome-Wide DNA Methylation Profiling as Frontline Diagnostics for Central Nervous System Embryonal Tumors in Hong Kong

**DOI:** 10.3390/cancers15194880

**Published:** 2023-10-07

**Authors:** Otto C. H. Tam, Ronnie S. L. Ho, Shing Chan, Kay K. W. Li, Tit-Leung Lam, Elaine T. Y. Cheung, Oi-Yee Cheung, Wilson W. S. Ho, Kevin K. F. Cheng, Matthew M. K. Shing, Dennis T. L. Ku, Brian H. Y. Chung, Wanling Yang, Godfrey C. F. Chan, Ho-Keung Ng, Anthony P. Y. Liu

**Affiliations:** 1 Department of Paediatrics and Adolescent Medicine, The University of Hong Kong, Pok Fu Lam, Hong Kong; ottotam@hku.hk (O.C.H.T.);; 2 Department of Pathology, Gleneagles Hospital, Wong Chuk Hang, Hong Kong; 3 Department of Anatomical and Cellular Pathology, Chinese University of Hong Kong, Sha Tin, Hong Kong; 4 Department of Pathology, Queen Elizabeth Hospital, Kowloon, Hong Kong; 5 Department of Neurosurgery, Queen Mary Hospital, Pok Fu Lam, Hong Kong; 6 Department of Neurosurgery, Hong Kong Children’s Hospital, Kowloon, Hong Kong; 7 Department of Paediatrics and Adolescent Medicine, Hong Kong Children’s Hospital, Kowloon, Hong Kong

**Keywords:** pediatrics, CNS tumors, methylation profiling, diagnostics

## Abstract

**Simple Summary:**

Genome-wide DNA methylation profiling has emerged as an important diagnostic tool that complements histopathology for characterizing central nervous system (CNS) tumors. The literature describing its application in Asian countries is limited. We report the feasibility and utility of adopting such profiling for children with diagnosed CNS tumors in Hong Kong. The study included samples of 97 patients with CNS embryonal or high-grade neuroepithelial tumors (HGNETs). In our DNA methylation-profiled cohort, 67% of results correlated with the diagnosis and assigned molecular subgroup and 12% of results led to revision/reassignment of diagnosis. Patient outcomes correlated significantly with methylation-based subgroups. In addition, novel clinical–histologic–molecular associations were described. Taken together, epigenomic profiling enabled clinically relevant refinement of disease classification of pediatric CNS tumors. The availability of such methodology in Asia sets the stage for international collaborations in molecularly driven trials.

**Abstract:**

This paper examines the link between CNS tumor biology and heterogeneity and the use of genome-wide DNA methylation profiling as a clinical diagnostic platform. CNS tumors are the most common solid tumors in children, and their prognosis remains poor. This study retrospectively analyzed pediatric patients with CNS embryonal tumors in Hong Kong between 1999 and 2017, using data from the territory-wide registry and available formalin-fixed paraffin-embedded tumor tissue. After processing archival tumor tissue via DNA extraction, quantification, and methylation profiling, the data were analyzed by using the web-based DKFZ classifier (Molecular Neuropathology (MNP) 2.0 v11b4) and t-SNE analysis. Methylation profiles were deemed informative in 85 samples. Epigenetic data allowed molecular subgrouping and confirmed diagnosis in 65 samples, verified histologic diagnosis in 8, and suggested an alternative diagnosis in 12. This study demonstrates the potential of DNA methylation profiling in characterizing pediatric CNS embryonal tumors in a large cohort from Hong Kong, which should enable regional and international collaboration in future pediatric neuro-oncology research.

## 1. Introduction

Central nervous system (CNS) tumors are the most common solid tumor type in children and have the highest mortality rates among various pediatric malignancies [1]. Therapeutic approaches for CNS tumors consist of maximal surgical resection with or without chemoradiation. Even with the availability of magnetic resonance imaging, improved neurosurgical methods, and the precise delivery of external beam radiation, treatment outcomes remain far from satisfactory [2]. CNS embryonal tumors, high-grade glioma, and ependymoma are entities with especially poor prognoses.

The past decade witnessed a rapid explosion in our knowledge of pediatric CNS tumors’ genomic, transcriptomic, and epigenomic features [3]. Since the 2016 revision to the World Health Organization (WHO) CNS Tumor Classification, molecular findings have been incorporated in the definition of various diagnostic categories [4]. Medulloblastoma, for example, has now been robustly stratified based on methylation profiling into wingless (WNT)-activated, Sonic hedgehog (SHH)-activated, Group 3, and Group 4, with group-specific cells of origin and pathway disruptions being correlated with histopathological variants, genomic alternations, clinical features, and survival. Ongoing trials have incorporated molecular stratification in order to allow personalized treatment in children with medulloblastoma. Similarly, other CNS embryonal tumors, notably, CNS primitive neuroectodermal tumor (CNS-PNET), atypical teratoid/rhabdoid tumor (ATRT), and pineoblastoma, have now been shown to be molecularly heterogeneous, particularly at the epigenetic level [5].

Although combined clinico-radiological and histopathological features will remain core to the classification of pediatric CNS tumors, the application of genome-wide DNA methylation profiling has evolved from a research tool to become a clinically relevant diagnostic platform. Histopathological grading schemes, while prognostically significant in certain entities, have been shown to demonstrate inter-observer variability, are often challenging to standardize [6], and for some entities are superseded by molecular features in predicting treatment outcome [7,8]. Therefore, methylation array profiling is increasingly being integrated into the clinical molecular pipeline for the upfront diagnosis of CNS tumors.

As much of the existing literature reflects the experience of leading research pediatric oncology institutes or national precision medicine programs, there is a need to consolidate the utility of methylation profiling in other settings based on real-world data, especially from Asia [9,10,11,12]. Herein, we report the feasibility and clinical utility of applying methylation profiling on a cohort of nearly 100 archival CNS embryonal tumor samples from a population-wide cohort in Hong Kong. As a single molecular assay, a methylation study is shown to facilitate molecular grouping, verification of diagnosis, and definition of novel associations in these patients.

## 2. Materials and Methods

### 2.1. Inclusion Criteria

Prior to 2019, pediatric patients with cancer in Hong Kong were managed in 1 of the 5 pediatric oncology units (Queen Mary Hospital, Prince of Wales Hospital, Queen Elizabeth Hospital, Tuen Mun Hospital, Princess Margaret Hospital), with data prospectively registered in the territory-wide registry hosted by the Hong Kong Paediatric Haematology/Oncology Study Group (HKPHOSG). In this study, we retrospectively identified patients with CNS embryonal tumors and associated diagnostic entities diagnosed between 1999 and 2017. The relevant diagnoses included medulloblastoma; CNS-PNET; pineal parenchymal tumors; embryonal tumor with multilayered rosettes (ETMR) and its variants; embryonal tumor, not otherwise specified (NOS); and high-grade neuroepithelial tumor (HGNET). Those with available formalin-fixed paraffin-embedded (FFPE) tumor tissue were included for analysis. This study was approved by the University of Hong Kong/Hospital Authority Hong Kong West Cluster Institutional Review Board (Reference Number: UW 19-078).

### 2.2. DNA Extraction and Methylation Profiling

Archival FFPE tumor tissue was retrieved, and DNA extraction was performed by using the QIAamp DNA FFPE Tissue Kit (Qiagen, Germantown, MD, USA) according to the manufacturer’s instructions. Extracted DNA was quantified via Qubit, and quality control was performed by using the qPCR-based Infinium HD FFPE QC Kit (Illumina, San Diego, CA, USA). DNA specimens fulfilling the quality control criteria (delta-Ct < 5) underwent bisulfite conversion using EpiTect Bisulfite Kit (Qiagen, Germantown, MD, USA) and were then used for DNA-methylation profiling with Infinium MethylationEPIC BeadChip (Illumina, San Diego, CA, USA) targeting 850,000 CpG sites in the genome (Centre for Genomic Sciences, HKU). The resulting raw Intensity Data files were analyzed with the web-based German Cancer Research Center (DKFZ) classifier (Molecular Neuropathology [MNP] 2.0 v11b4 https://www.molecularneuropathology.org/mnp accessed on 1 December 2022), with each sample being assigned a molecular class and a corresponding confidence score. In parallel, unsupervised t-distributed Stochastic Neighbor Embedding (t-SNE) analysis was performed to compare the study cases with a publicly available CNS tumor reference cohort based on 2801 samples [12]. Molecular calls were based on the results from both analyses and further interpreted according to the clinical contexts. Copy-number profiles derived from methylation data by using the conumee package (Bioconductor) were inspected manually.

### 2.3. Statistical Analysis

Overall survival (OS) was defined as the interval from diagnosis to the date of death or last follow-up for survivors; progression-free survival (PFS) was defined as the interval from the date of diagnosis to the date of the first event (i.e., disease progression, disease recurrence, second malignant neoplasm, or death from any cause) or to the date of last follow-up for patients without events. Survival estimates were reported using the method of Kaplan and Meier. The log-rank test was used to compare survival among groups, and Cox regression was used for multi-variate analyses. All reported *p*-values were two-sided and considered to be significant at a value < 0.05.

## 3. Results

### 3.1. Demographic and Clinical Features

Samples from 97 patients (59 male and 38 female) were profiled. The median age at diagnosis was 5.7 years (range: 0.6–22.2 y), and the median duration of follow-up was 4.6 years (range: 0–20.6 y). The primary tumor location was cerebellar/posterior fossa in 71, cerebral in 15, pineal in 7, sellar/parasellar in 2, ventricular in 1, and spinal in 1. Metastasis was documented in 15% of cases. Among patients for whom outcome data were available (*n* = 85), an event occurred in 49 patients (47 = tumor related).

### 3.2. Tumor Specimen and Quality Control

DNA was extracted from FFPE slides (*n* = 75), scrolls (*n* = 3), or a combination of both (*n* = 19). The sectioning thickness was 8–10 µm, and 3–30 sections were used per specimen. In seven samples, macrodissection was required to enrich for tumor material due to low tumor content. The median quantity of total DNA extracted, as determined by Qubit, was 490 ng (range: 12.5–7290 ng). Up to 500 ng of DNA was submitted for methylation array. Despite the variation in DNA concentration, real-time PCR following the Illumina FFPE QC protocol deemed all samples to be suitable for methylation array profiling (Delta-Ct < 5 compared to the quality control template).

### 3.3. Histopathologic-Molecular Correlation and Clinical Utility

**Figure 1:**
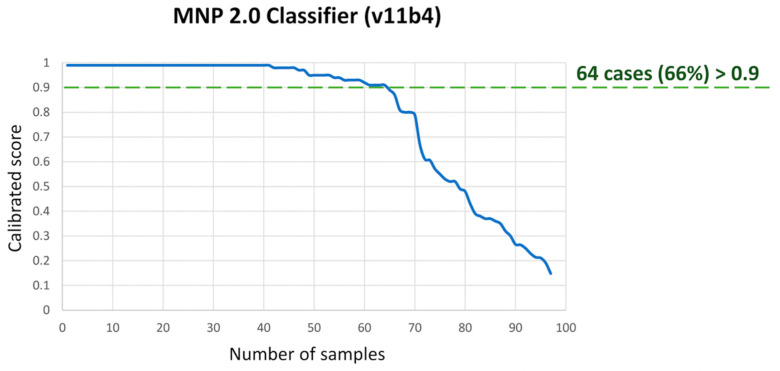
Summary of calibrated confidence score for assigned methylation class on the MNP 2.0 Classifier for our cohort.

**Figure 2:**
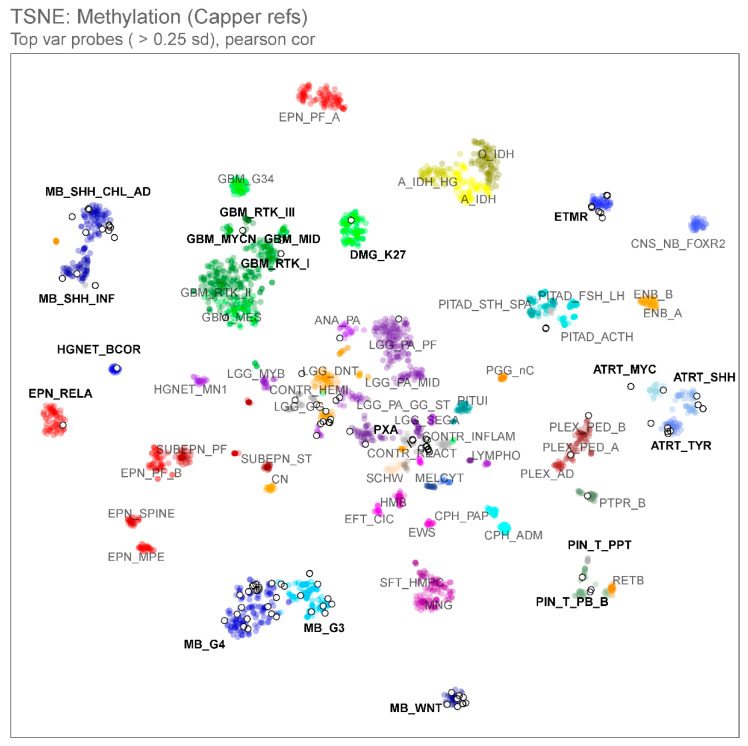
t-distributed stochastic neighbor embedding (t-SNE) analysis comparing the methylation profiles of our study cohort (*n* = 97, open white circles) and publicly available central nervous system (CNS) tumor reference entities (*n* = 2801, colored circles, Capper et al., 2018 [12]), indicating the association between samples and known molecular subclasses of CNS tumors with infrequent outliers.

The original pathologic diagnoses included medulloblastoma (*n* = 65), ATRT (*n* = 9), pineal parenchymal tumor (*n* = 8), ETMR (*n* = 6), HGNET (*n* = 4), CNS-PNET (*n* = 4), and choroid plexus tumor (*n* = 1). When assessed on the random-forest classifier by MolecularNeuropathology 2.0 (version 11b4), calibrator scores >0.9 were achieved in 64 samples (66%), and scores >0.6, in 73 samples (75%) (Figure 1). Complementing the above with unsupervised t-SNE analysis based on the CNS tumor references (*n* = 2801) by Capper et al. [12], methylation profiles were deemed informative in 85 samples (88%) (Figure 2). The methylation-based assignments are summarized in Table 1.

**Figure 3:**
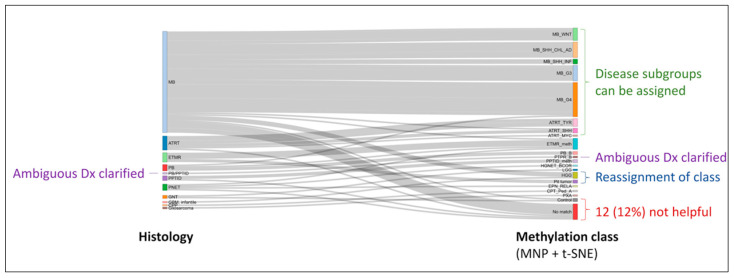
Alluvial plot depicting the molecular heterogeneity within histologic entities as revealed by methylation profiling. Clarification of ambiguous diagnosis (Dx) and reassignment of diagnostic classes are additional advantages of incorporating such assays into the diagnostic pipeline.

Epigenetic data allowed molecular subgrouping and confirmed diagnosis in 65 samples (67%), verified histologic diagnosis in 8 samples (8%), and suggested alternative diagnosis in 12 samples (12%) (Figure 3). For the remainder of the samples, four were molecularly similar to control references, suggestive of non-neoplastic cell contamination, but the other eight samples did not cluster with any references on the classifier or t-SNE.

**Figure 4:**
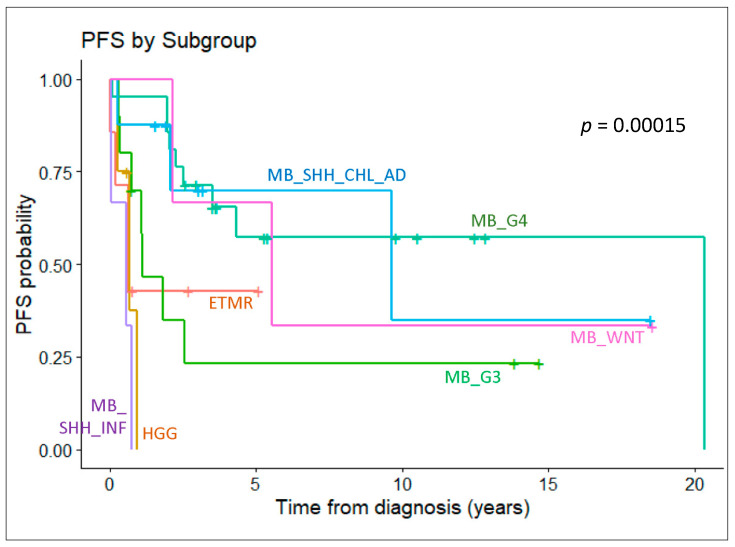
Survival analysis depicting the progression-free survival of patients with key molecular entities defined by methylation profiling. Drastic differences in outcome are observed among those with different subgroups of medulloblastoma.

Patient survival differs significantly when stratified by molecular diagnosis (Figure 4). Entities with better outcomes include medulloblastoma of the WNT, SHH (children/adult), and Group 4 subgroups; those with inferior outcomes include medulloblastoma of the SHH (infant) and Group 3 subgroups, ETMR, and high-grade glioma. Although these entities had been treated by using a uniform approach (including glioma that was misdiagnosed as an embryonal tumor), a molecularly stratified treatment strategy is obviously required in the future.

### 3.4. Methylation Profiling Unveils Novel Histologic-Molecular Entities

The 12 samples with mismatches between histologic and molecular diagnoses were further reviewed and examined, if tumor material was available.

**Figure 5:**
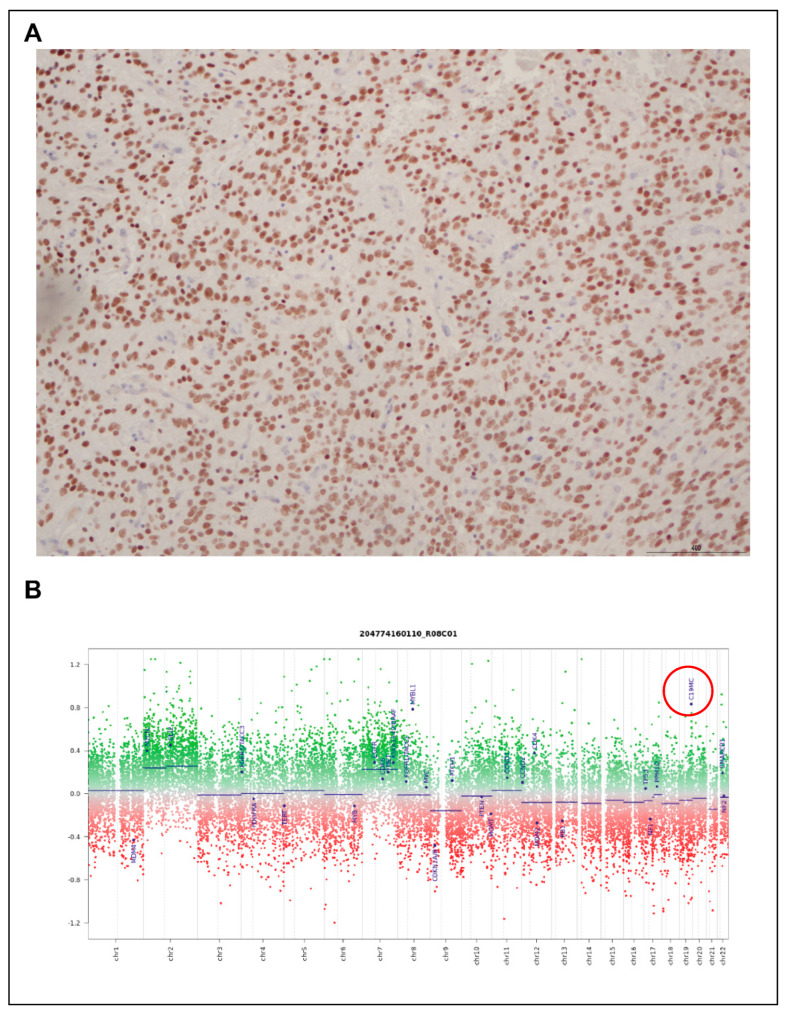
Orthogonal validation of methylation clustering findings. (**A**) BCOR IHC positivity in a sample reclassified as HGNET-BCOR; (**B**) copy-number amplification of *C19MC* (red circle) in a molecularly diagnosed ETMR.

Among the three samples classified historically as CNS-PNET, methylation profiling suggested the molecular diagnosis of the high-grade neuroepithelial tumor with BCOR alteration (HGNET-BCOR), ETMR, and ependymoma with RELA-fusion. Additional immunohistochemistry testing was performed on the sample with the molecular diagnosis of HGNET-BCOR, confirming BCOR expression (Figure 5A), whereas *C19MC* amplification was observed on the methylation-derived copy-number plot for the molecular ETMR sample (Figure 5B), supporting the diagnosis.

For two patients with ETMRs (histological variants—ependymoblastoma and medulloepithelioma) of the sellar region, methylation profiles had no high-quality match on the classifier but were closely associated with pituitary tumor references by t-SNE (previously described in detail [13]). Next-generation sequencing was performed on paired tumor-germline samples for both patients, revealing mutations in *DICER1*. Such information prompted further evaluation of the relationship among these two samples and the rare, DICER1-associated sellar tumor known as pituitary blastoma that exclusively occurs in children younger than 2 years. As previously reported, clustering analysis including additional reference data from pituitary blastoma, pituitary adenoma, and normal pituitary gland confirmed the similarity between our study samples and reference pituitary blastoma. Unexpectedly, one sample also harbored amplification of *C19MC*, typically seen only in cases of ETMR. Further microRNA profiling of the study samples confirmed dysregulated mature microRNA generation leading to a 3p/5p imbalance, characteristic of DICER1-associated tumors. With one of the patients developing the tumor at the age of 8 years after a history of leukemia, our study of these two cases expanded the clinical and molecular spectrum of pituitary blastoma.

**Figure 6:**
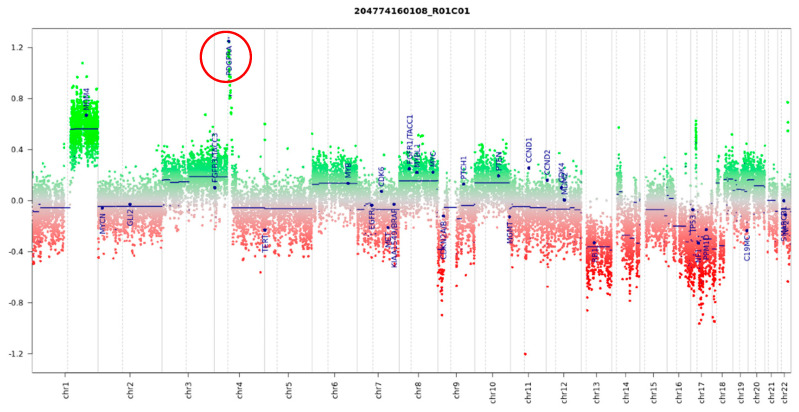
Copy-number analysis based on methylation data indicates a high level of *PDGFRA* amplification (red circle) in a sample reclassified as high-grade glioma.

In three patients, initial histologic diagnoses of medulloblastoma were challenged by methylation assignment to high-grade glioma subgroups, namely DMG-K27, GBM-RTKIII, and GBM-MID. Further characterization confirmed H3K27M mutation, *MYCN* amplification, and *PDGFRA* amplification in these respective samples (Figure 6). Additionally, the tumors in all three patients displayed an aggressive clinical course refractory to chemoradiation, atypical of what would be expected for medulloblastoma. The review of the MRIs of these patients confirmed cerebellopontine angle primaries, and tumor sequencing data indicate *TP53* mutations in all.

## 4. Discussion

Embryonal tumors are the most prevalent malignant CNS tumors in young children. They represent aggressive WHO Grade IV tumors with a fair outcome despite treatment with intense multimodal treatment. For decades, the various subtypes of CNS embryonal tumors have been considered to be biologically similar and were treated with the same protocols. As discussed, recent epigenomic studies have concluded otherwise. With the WHO CNS Tumor Classification 2021 recommendation that methylation profiling be incorporated into routine diagnostics, it is timely to evaluate the utility of such a tool in our local, real-world setting [14].

Specimen availability and quality represent some of the most commonly encountered hurdles to molecular assays. Methylation arrays enable the generation of quality data based on FFPE tissue-derived DNA, which is in stark contrast to transcriptomic studies, which largely rely on the availability of snap-frozen samples for preservation of RNA quality [15]. Our experience confirms that even with DNA quantities much lower than the input amount recommended by the manufacturer (500 ng) as determined by fluorometric assays, it is worthwhile to proceed with qPCR-based QC and the downstream experiment. In our cohort, 88% of data generated clustered with established tumor entities. Although it is possible that suboptimal classification might be related to sample quality and purity issues, our recent experience with the updated MNP classifier versions suggests that suboptimal classification may be contributed to by the classifier algorithms. Therefore, it is likely that the success rate with methylation studies for tumor classification will continue to improve with time using similar chemistry but with enhanced bioinformatic pipelines.

Methylation-based classification has been crucial to the accelerated discovery of novel CNS tumor groups, particularly in the case of CNS embryonal tumors. In addition to the four medulloblastoma groups described, SHH tumors have since then been divided into four subtypes and Group 3/4 in to eight subtypes [16]. Pineoblastomas are described as having four molecular subgroups in the latest WHO classification and ATRTs have been divided into the tyrosinase (TYR), MYC, and SHH subgroups [17,18]. Similarly, clinically relevant subclasses have been described in other CNS tumor entities, impacting patient management and clinical trial design. Although orthogonal biomarkers, such as those used in IHC panels, are being validated for the diagnosis of certain methylation-based subgroups [19], these are not available for all entities, and their diagnostic accuracy falls short of that of methylation array data. This also imposes a great burden on individual laboratories in terms of the effort and cost required to acquire and maintain such surrogate tests for the increasing number of novel entities for which case numbers are low. Furthermore, as the use of next-generation DNA sequencing alone is incapable of identifying many of these tumor entities, it might be prudent to consider an epigenotype-driven diagnostic workflow, which could be time-, cost- and tissue-saving in comparison with the conventional approach.

Although initially used as a research tool, methylation arrays should now be adopted by clinical laboratories for use in diagnosing CNS and other solid tumors. We demonstrate that 12% of diagnoses had to be revised after completion of the methylation study, in keeping with other reported cohorts in which approximately 10% of diagnoses made by expert neuropathologists had to be reviewed after the availability of epigenetic data [9,10,12]. The rare entities/associations described, namely, atypical pituitary blastomas and pediatric cerebellar high-grade glioma with *TP53* mutations, highlight the challenges of diagnosing CNS tumors via conventional pipelines [13].

This study’s main limitation is that the diagnostic accuracy remains dependent on the quality, size, and complexity of the reference cohort. The analysis associating treatment outcomes with methylation groups may have been hampered by the improvement in diagnostic tools, imaging, and supportive care that occurred over the study period. Another limitation is that certain entities, such as CNS germ cell tumors, are underrepresented in the existing literature as their incidence is relatively low in the Western population [20]. Continued research and conglomeration of data from multi-national collaborations is likely required to further enhance the accuracy of such epigenetic-based algorithms in histologic diagnosis. In fact, the availability of molecular data will facilitate twinning programs with regions where the exchange of tumor material for pathology review would be cumbersome. Nonetheless, it should also be emphasized that methylation profiling cannot supplant the expert evaluation by neuropathologists which will also be involved in an integrated interpretation of clinical information, histomorphology, immunohistochemical profiles, and other targeted molecular data [4].

## 5. Conclusions

Taken together, we demonstrated the feasibility and utility of performing DNA methylation profiling to characterize pediatric CNS embryonal tumors in a large cohort of patients from Hong Kong. Our study is one of the largest in Asia and sets the stage for regional and international collaboration for clinical research in pediatric neuro-oncology.

## Figures and Tables

**Table 1:** Summary of methylation-based assignment of central nervous system embryonal tumors included in study.

Entity	Subgroup	*n*
Total		97
Medulloblastoma		52
	Wingless-activated	8
	Sonic hedgehog-activated (SHH) (infant)	3
	SHH (children/adult)	10
	Group 3	10
	Group 4	21
Atypical teratoid/rhabdoid tumor		9
	TYR	5
	SHH	3
	MYC	1
Pineal parenchymal tumors		6
	Pineoblastoma	3
	Pineal parenchymal tumor of intermediate differentiation	2
	Papillary tumor of pineal region	1
Embryonal tumor with multi-layered rosettes		7
Pituitary blastoma		2
High-grade neuroepithelial tumor, BCOR-altered		1
High-grade glioma		4
Low-grade glioma/ganglioglioma		1
Pleomorphic xanthastrocytoma		1
Ependymoma, RELA-fusion positive		1
Choroid plexus tumor		1
Control		2
No match		10

## Data Availability

The data presented in this study are available from the corresponding author upon reasonable request.
